# Response in Patients With Persistent Pelvic Pain to Motor Imagery Through Auditory or Visual Input—A Pilot Randomized Trial

**DOI:** 10.1155/prm/1412626

**Published:** 2025-02-10

**Authors:** Borja Perez-Dominguez, Alba Arce-Elorza, Isabel Rubio-Garcia, Esther Diaz-Mohedo

**Affiliations:** ^1^Department of Physiotherapy, University of Valencia, Valencia, Spain; ^2^Department of Physiotherapy, University of Málaga, Málaga, Spain

**Keywords:** clinical trials, imagery, pain, pelvic pain, physical therapy modalities, randomized

## Abstract

**Purpose:** This study evaluates the response to a motor imagery intervention using visual or auditory inputs in patients with persistent pelvic pain. A secondary objective is to assess how patients' mental visualization capacity influences intervention outcomes.

**Methods:** Forty patients diagnosed with persistent pelvic pain were enrolled in a randomized trial with six motor imagery sessions over 2 weeks. Patients were assigned to interventions delivered through images or audio recordings. Pain intensity, attention to pain, and the ability to mentally visualize and perceive movements were assessed.

**Results:** Participants receiving auditory stimulus–based interventions showed a nonsignificant reduction in pain intensity (from 7.1 points [SD: 1.9] to 6.1 points [SD: 2.4]; *p*=0.091), while those in the visual input group experienced no change. Attention to pain improved in the visual group (from 30.2 points [SD: 6.2] to 27.6 points [SD: 6.8]; *p*=0.194), whereas it remained stable in the auditory group. Importantly, the participants' ability to mentally visualize and perceive movements did not significantly impact the outcomes.

**Conclusions:** Auditory motor imagery appears to be a promising, less intrusive approach for managing persistent pelvic pain, with home-based interventions showing potential where access to conventional care is limited. This study highlights the importance of personalized motor imagery approaches, demonstrating superior efficacy for auditory interventions compared to visual ones. Limitations include a brief intervention period and recruitment challenges, yet motor imagery remains a viable therapeutic option.

**Trial Registration:** ClinicalTrials.gov identifier: NCT06343649

## 1. Introduction

Persistent pelvic pain (PPP) is a recurring pain experienced in structures associated with the pelvis in both men and women. This condition is frequently linked to adverse cognitive, behavioral, sexual, and emotional outcomes, along with symptoms indicative of lower urinary tract syndrome, as well as sexual, bowel, pelvic floor, or gynecological dysfunctions. To be classified as persistent pain, the pain must be continuous or recurrent for a minimum of 3 months. In instances of cyclical pain, a more extended period exceeding 6 months may be deemed more appropriate [1].

The nervous system is dynamic, with neurons becoming sensitized as nociceptive input persists, leading to alterations in cortical representation. These changes can contribute to the complexities of pain perception [2]. Imaging studies show that pain does not originate from a singular “pain center;” rather, multiple cortical areas can be activated, reflecting variability in pain experiences. Chronicity heightens synaptic efficacy within the pain neuromatrix, making even smaller inputs sufficient to trigger pain [3].

Given the challenges of managing PPP, pharmacological interventions are often prioritized as the initial treatment, while surgical options are reserved for the last resort, and both approaches have limited success. A promising alternative is graded motor imagery (GMI), a brain-based treatment designed to engage different brain regions in a controlled manner [4]. GMI consists of sequential phases that include implicit motor imagery tasks and motor imagery rehearsal.

The effectiveness of GMI relies on adhering to this sequence, which allows patients to engage in activities without provoking pain flare-ups [5]. Progression through these phases can be tailored to individual pain levels and abilities to visualize movements, which is crucial for successful outcomes [6]. This capacity is typically assessed through self-administered tools such as the movement imagery questionnaire–revised (MIQ–R), capturing the subjective experience of mental imagery in motor tasks [7].

In a study conducted by Ikeda et al. in 2012 [8], the response of the brain to various evoked potentials (visual and auditory) was evaluated using transcranial magnetic stimulation. The findings revealed that visual stimuli elicit a more pronounced activation in the brain compared to auditory stimuli. Given that the motor imagery phase usually involves the visualization of images, an alternative therapeutic approach utilizing auditory inputs could be considered less intrusive and potentially safer for patients with PPP.

Hence, the primary objective of this study is to evaluate the response to a motor imagery intervention conducted using either visual or auditory inputs in patients with PPP. A secondary aim is to assess how the participants' capacity to mentally visualize and perceive movements influences the outcomes of the motor imagery intervention.

## 2. Methods

### 2.1. Design

This study is a randomized clinical trial conducted to assess the effects of a motor imagery intervention applied using visual or auditory inputs in patients suffering from PPP. The protocol for this clinical trial was approved by the Ethics Committee of the University of Malaga, Spain (reference number: CEUMA: 187-2023-H). The study has been carried out in accordance with the Code of Ethics of the World Medical Association, and every participant was given a brief explanation of the study procedure prior to providing written consent. To ensure comprehensive and transparent reporting, this study adheres to the CONSORT guidelines. The completed CONSORT checklist is provided as supporting information (Supporting Information 1).

### 2.2. Participants

Recruitment took place during the month of October 2023 through dissemination by the members of the research team on their social media accounts and collaboration with a Spanish association for patients suffering from pelvic pain (ADOPEC). Potential participants were asked to complete an online form providing demographic information and completing a validated instrument for discriminating chronic pelvic pain, the chronic pelvic pain questionnaire (CPPQ-Mohedo) [9].

Inclusion criteria were as follows: (1) Adult < 18 years, (2) suffering PPP for a period of over 6 months, and (3) scoring at least a 6 in the CPPQ-Mohedo questionnaire. Participants were excluded if they had a medical condition that explained the presence of pain reasonably.

### 2.3. Interventions

Participants received a total of six sessions of motor imagery for 2 weeks. Exercises were developed by the members of the research team, experienced physical therapists who specialize in pelvic floor disorders, according to a gradual exposure therapy program.

Participants were allocated to a group that received these sessions through visual inputs, in the form of images, or a group that received these sessions through auditory inputs, in the form of audio recordings. Both groups followed the same gradual exposure therapy program designed to represent a gradual progression of potentially painful activities. Members of the research team in charge of the intervention sent via email the exercises on alternate days. To ensure adherence to the program, each group had a blinded researcher responsible for sending the exercises and monitoring participant compliance. Researchers maintained regular contact with participants during the intervention to ensure that the program was followed correctly.

#### 2.3.1. Motor Imagery Through Images

Participants allocated in the group receiving the intervention through visual input received an email with a detailed explanation of the exercise they had to perform accompanied by carefully selected images. Participants were instructed to read the exercise, find a comfortable position, and concentrate to imagine themselves in the proposed scenario. Then, participants were instructed to visualize and concentrate on images that were related to the exercise and repeat the mental observation task. Exercises included sitting on a stool or a rigid chair, being assessed by a clinician, performing strenuous exercise, or receiving sexual stimulation.

Images were carefully selected by members of the research team in charge of the intervention. To ensure participants of each gender received images they could relate to, images representing males and females were included. The selection of images for male participants was conducted by retrieval from the internet, and the selection of images for female participants was conducted using a compilation that includes a variety of views of pelvises in different situations (©The Vulvar Image Collection Katie Kelly PT, 2022).

#### 2.3.2. Motor Imagery Through Audio

Participants allocated in the group receiving the intervention through auditory input received an email containing an audio file with a recording of the exercise they had to perform. Participants were instructed to find a comfortable place with no external distractions and were encouraged to use headphones. The auditory sessions included verbal instructions guiding participants through imagined scenarios, mirroring the exercises provided to the visual input group.

Audios were recorded and edited by members of the research team, following the same gradual exposure program, and contained guided sessions where the patients were given verbal instructions to follow through imagination.

### 2.4. Outcomes

Two members of the research team, blinded to participant allocation, conducted the assessments a week before the intervention started and a week after it ended. Participants were asked to complete an online form containing self-administered assessment instruments. Demographic characteristics included age, sex, participant's marital status, and if the participant was receiving or not active treatment. If missing values were found, these will be addressed through imputation to ensure the preservation of the sample size and statistical power.

Primary outcomes included pain intensity and participant's attention to pain. Pain intensity was assessed through the numerical rating scale (NRS) [10], an instrument that requires the patient to rate their pain intensity on a 0–10 scale, where 0 is no pain and 10 is the worst pain imaginable. Higher scores indicate worse outcomes. Participant's attention to pain was assessed through the Spanish version of the pain vigilance and awareness questionnaire (PVAQ) [11], a 16-item questionnaire, where each item is scored as a six-point Likert scale that ranges from 0 (*never*) to 5 (*always*), resulting in a sum score between 0 and 80. Higher scores indicate worse outcomes.

As a secondary outcome, the participant's ability to mentally see and feel movements was assessed. To conduct this assessment, the Spanish version of the MIQ–R [12] was used. This instrument has been validated as one of the most useful when assessing motor imagery [13, 14]. This instrument includes four movements and participants are asked to rate on a scale ranging from 1 to 7 based on how easy it is to visualize and feel each movement, 1 being very difficult to see/feel and 7 being very easy to see/feel. The score ranges from 8 to 56 points, with higher scores resulting in a better ability to mentally see and feel movements.

### 2.5. Statistical Analysis

Given the exploratory nature of the study, a convenience sample of at least 40 participants was recruited, aiming to inform adjustments for a larger-scale study. A member of the research team, who was blinded to assessment and intervention, randomized participants using a sealed opaque envelope system with a 1:1 ratio to either a group that received the intervention through visual inputs or a group that received the intervention through auditory inputs. Participants were blinded, as were the researchers in charge of data collection and analysis.

Analyses were conducted using SPSS (SPSS Inc, Chicago, Illinois) software, in its 28.0 Version for MacOS. Descriptive statistics were reported as mean and standard deviation (SD) for quantitative outcomes and frequencies for qualitative outcomes. The normal distribution of data was checked through the Shapiro–Wilk test.

Pain intensity and participant's attention to pain were analyzed employing a mixed-model two-way ANOVA to assess both between-group factors (visual or auditory inputs) and within-group factors (change over time). These outcomes were subsequently used to establish a correlation with the participant's ability to mentally see and feel movements, employing Pearson's correlation coefficient due to the inherent nature of the outcomes. Potential baseline differences between the groups were conducted through statistical comparisons of baseline data using independent samples *t*-tests for continuous variables and chi-square tests for categorical variables.

## 3. Results

**Figure 1:**
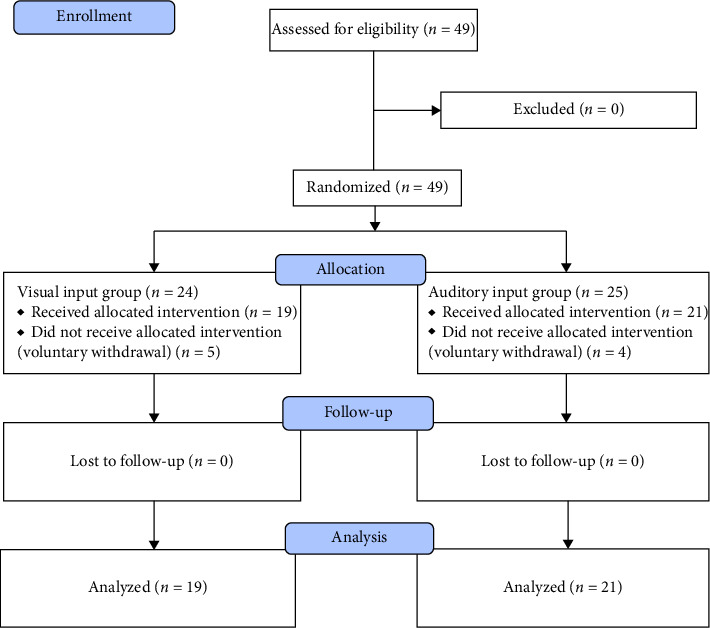
Participant's flowchart.

Initially, 49 participants were enrolled and randomized, with 24 allocated to the cohort exposed to the intervention via visual input and 25 to the cohort receiving the intervention through auditory input. Subsequently, 5 participants from the visual input group and 4 from the auditory input group voluntarily withdrew from the study postallocation. This led to a total of 40 participants who were initially evaluated for primary outcomes. Notably, none of these 40 participants were lost to follow-up, and all successfully completed the assessments upon the conclusion of the intervention. A summary of participant flow can be found in Figure 1. Baseline data comparison results indicated no significant differences between the groups in terms of demographic or baseline pain–related variables.

### 3.1. Characteristics of the Sample

The baseline demographic data of the sample is summarized in Table 1. The sample's overall mean age was 44.6 years (SD: 12.3), and participants were majorly women (82.5%), married (45%), who received active treatment (72.5%). Participants scored an overall of 18.6 points (SD: 4.2) on the CPPQ-Mohedo and 37.5 points (SD: 11.1) on the MIQ–R. Data were normally distributed.

### 3.2. Results on the Analysis of Motor Imagery Through Visual or Auditory Input

**Figure 2:**
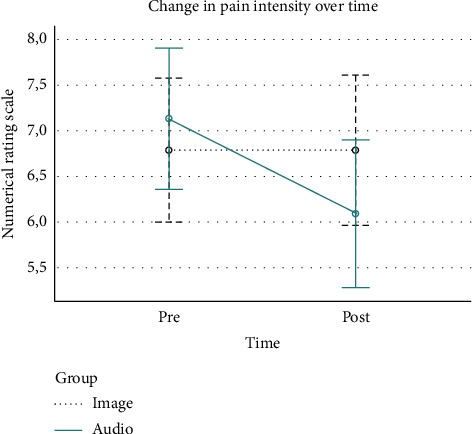
Change over time in pain intensity.

The results of the ANOVA analysis are summarized in Table 2. Pain intensity in participants allocated to the group receiving the intervention through visual inputs remained the same. However, pain intensity improved in participants allocated to the group receiving the intervention through auditory inputs (from 7.1 points [SD: 1.9] to 6.1 points [SD: 2.4]), even though this improvement was nonsignificant (*p*=0.091). A graphical representation of these results can be found in Figure 2.

**Figure 3:**
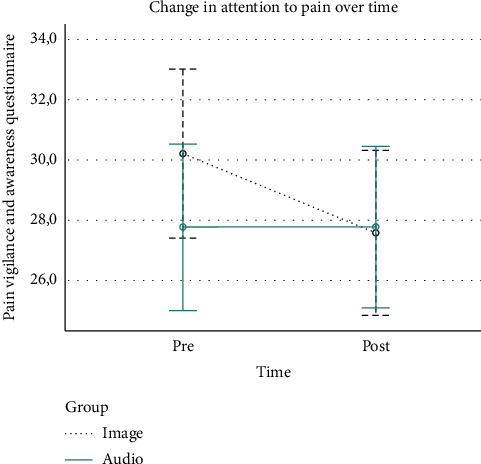
Change over time in attention to pain.

Levels of attention to pain improved nonsignificantly (*p*=0.194) in participants allocated to the group receiving the intervention through visual inputs (from 30.2 points [SD: 6.2] to 27.6 points [SD: 6.8]). However, levels of attention to pain remained the same in the group receiving the intervention through auditory inputs. A graphical representation of these results can be found in Figure 3.

### 3.3. Results on the Influence of Participant's Ability to Mentally See and Feel Movements

The results for the correlation analysis between the participant's ability to mentally see and feel movements and results from the intervention are summarized in Table 3. There are no significant levels of correlation between the ability to mentally see and feel movements and any outcomes in any group.

## 4. Discussion

This research entails a randomized trial designed to compare the responses of patients with PPP when subjected to a motor imagery intervention delivered either visually or auditorily, with a focus on pain intensity and attention to pain. A secondary aim was to evaluate the impact of participants' capacity to mentally visualize and perceive movements on the outcomes. The findings reveal a reduction in pain intensity among participants undergoing motor imagery through auditory inputs, although this improvement did not reach statistical significance. Moreover, attention to pain improved for those undergoing motor imagery through visual inputs, with a similar lack of statistical significance. Correlation analysis indicates that the participants' ability to mentally visualize and perceive movements does not significantly influence the responses observed across all groups.

One of the key strengths of this study is its focus on an underresearched population, addressing a gap in the literature regarding the management of PPP. The randomized design enhances the robustness of the findings and provides a foundation for future research in this area. GMI is frequently misunderstood by participants who exhibit reluctance to engage in an intervention that does not directly address the specific region of pain they experience. Consequently, this misconception contributes to low compliance levels and elevated dropout rates, as evidenced in this study where nine participants opted to withdraw after their allocation. It is crucial to acknowledge the significance of this phenomenon, as the participation rate proves to be pertinent, necessitating sustained motivation from participants throughout the entirety of the intervention [15].

Our study aims to evaluate whether auditory inputs are less likely to trigger pain flare-ups compared to visual inputs during a motor imagery intervention. Prior literature [16, 17], supports the use of auditory inputs in pain management, where patients with pelvic pain experienced significant reductions in pain intensity following guided imagery recordings. This study contributes to this growing body of evidence by demonstrating the potential of auditory motor imagery interventions in this specific population.

In recent times, there has been a notable rise in the recognition of home-based applied therapies for the comprehensive management of individuals grappling with persistent pain. Given the constraints imposed on patients' access to conventional treatment modalities, particularly during events such as the global pandemic a few years ago, the imperative to devise alternatives that overcome these challenges has become evident. While home-based motor imagery interventions have gained traction as a therapeutic approach aimed at enhancing constructs such as functional performance [18, 19], their focus on pain and pain-related conditions has been limited. Our study is groundbreaking in its endeavor to introduce home-based interventions within a population experiencing persistent pain, presenting promising results that could prove valuable for both clinicians and patients worldwide. By facilitating access to effective pain management strategies, this research aligns with current trends toward telehealth and remote therapy options.

This study is not exempt from limitations. First, the intervention's short duration, spanning only 2 weeks, may have been insufficient to elicit significant changes. Further investigations are warranted to explore whether extending the intervention period yields more substantial improvements. In addition, the characteristics of our sample significantly influence the obtained results, as larger samples have the potential to yield more robust findings [20]. However, recruiting participants posed challenges due to the difficulty of conducting studies in an underdiagnosed condition, with participants generally displaying reluctance toward alternative treatments. Notably, participant compliance with the program was not documented in this study. While traditional motor imagery sessions are typically conducted face-to-face with a clinician present [5], our study employed online sessions, offering participants independence. In addition, none of the interventions used in this study activated the feared pain “flare-ups” that authors have described previously [21].

Another limitation relates to the standardized approach of our intervention. While this allowed us to assess the overall effect on a group level, a more individualized program, tailored to each participant's specific psychological and physical needs, could potentially be more effective. Despite this standardization, participants were given the freedom to revisit sessions or delay progression until they felt ready, which may have mitigated some of the limitations associated with a rigid protocol.

In summary, this research underscores the potential of auditory motor imagery as a viable option for managing PPP and highlights the importance of accessibility in therapeutic interventions. Future studies should continue to explore these innovative approaches, tailoring them to individual needs while assessing their efficacy in larger, more diverse populations.

The program was conducted exclusively in an online format, thereby improving accessibility for women who face barriers in accessing a specialized physiotherapist or expressing reservations about participating in conventional face-to-face therapy. Numerous factors, including experiences of shame, societal taboos, or embarrassment [22], contribute to the reluctance of many patients with PPP to seek assistance. Online therapeutic interventions present a promising solution [23].

## 5. Conclusion

This study suggests that auditory motor imagery may be an effective and minimally intrusive intervention for managing PPP. The findings indicate the potential value of home-based interventions in pain management, particularly in settings where access to traditional healthcare is limited. In addition, this study underscores the importance of considering individual patient preferences in motor imagery interventions, with auditory approaches showing promise compared to visual ones. However, given the limitations of our study, further research is needed to confirm these findings, explore long-term outcomes, and refine personalized motor imagery strategies for clinical application.

## Figures and Tables

**Table 1:** Baseline demographic characteristics of the participants.

	Visual input (*n* = 19)	Auditory input (*n* = 21)	Total (*n* = 40)
Age (years [SD])	45.2 (14.1)	44.1 (10.7)	44.6 (12.3)
Sex			
Men	7 (36.8%)	0 (0%)	7 (17.5%)
Women	12 (63.2%)	21 (100%)	33 (82.5%)
Marital status			
Single (unpartnered)	4 (21.1%)	5 (23.8%)	9 (22.5%)
Single (partnered)	4 (21.1%)	9 (42.9%)	13 (32.5%)
Married	11 (57.9%)	7 (33.3%)	18 (45%)
Receives treatment			
Yes	13 (68.4%)	16 (76.2%)	29 (72.5%)
No	6 (31.6%)	5 (23.8%)	11 (27.5%)
CPPQ (points [SD])	17.8 (3.9)	19.2 (4.5)	18.6 (4.2)
MIQ–R (points [SD])	38.5 (9.3)	36.5 (12.7)	37.5 (11.1)

Abbreviations: CPPQ, chronic pelvic pain questionnaire; MIQ–R, movement imagery questionnaire–revised; SD, standard deviation.

**Table 2:** Results of the mixed-design two-way repeated measures ANOVA.

Outcome	Group	Mean (SD)		Time, *p* value	Effect size	Group-time, *p* value	Effect size
		Pre	Post				
NRS (points [SD])	Visual	6.8 (1.9)	6.8 (1.4)	*F* = 2.979	*η*_*p*_^2^ = 0.060	*F* = 2.979	*η*_*p*_^2^ = 0.060
	Auditory	7.1 (1.9)	6.1 (2.4)	*p*=0.091		*p*=0.091	

PVAQ (points [SD])	Visual	30.2 (6.2)	27.6 (6.8)	*F* = 1.739	*η*_*p*_^2^ = 0.036	*F* = 1.739	*η*_*p*_^2^ = 0.036
	Auditory	27.8 (7.4)	27.8 (6.5)	*p*=0.194		*p*=0.194	

Abbreviations: NRS, numerical rating scale; PVAQ, pain vigilance and awareness questionnaire; SD, standard deviation.

**Table 3:** Results of the correlation analysis between results and participant's ability to mentally see and feel movements.

Outcome		Visual input		Auditory input	
		Pearson's correlation index	*p* value	Pearson's correlation index	*p* value
MIQ–R	NRS	0.030	*p*=0.888	−0.198	*p*=0.343
	PVAQ	0.167	*p*=0.434	−0.066	*p*=0.753

Abbreviations: MIQ–R, movement imagery questionnaire–revised; NRS, numerical rating scale; PVAQ, pain vigilance and awareness questionnaire.

## Data Availability

The data that support the findings of this study are available from the corresponding author upon reasonable request.

## Supporting Information

Supporting Information 1: A completed CONSORT checklist detailing the study's adherence to CONSORT reporting guidelines.
